# A novel principle to localize the sensitivity of waveform tomography using wave interferences at the observation boundary

**DOI:** 10.1038/s41598-021-01199-1

**Published:** 2021-11-11

**Authors:** Shohei Minato, Ranajit Ghose

**Affiliations:** Department of Geoscience and Engineering, Delft University of Technology, Stevinweg 1, 2628 CN Delft, The Netherlands

**Keywords:** Acoustics, Geophysics, Seismology, Mathematics and computing

## Abstract

When using waveform tomography to perform high-resolution imaging of a medium, it is vital to calculate the sensitivity in order to describe how well a model fits a given set of data and how the sensitivity changes with the spatial distribution of the heterogeneities. The traditional principle behind calculating the sensitivity—for detecting small changes—suffers from an inherent limitation in case other structures, not of interest, are present along the wave propagation path. We propose a novel principle that leads to enhanced localization of the sensitivity of the waveform tomography, without having to know the intermediate structures. This new principle emerges from a boundary integral representation which utilizes wave interferences observed at multiple points. When tested on geophysical acoustic wave data, this new principle leads to much better sensitivity localization and detection of small changes in seismic velocities, which were otherwise impossible. Overcoming the insensitivity to a target area, it offers new possibilities for imaging and monitoring small changes in properties, which is critical in a wide range of disciplines and scales.

## Introduction

Waveform tomography is an important approach to resolve unknown heterogeneities in a variety of materials and scales. For example, waveform tomography using acoustic, electromagnetic or seismic waves is used for in-vivo medical imaging to identify tumors^1,2^, for in-situ seismological monitoring of geological materials (e.g., rock, soil, ice, fluid)^3–5^, and for health monitoring of civil engineering structures (e.g., metal and concrete)^6^. Waveform tomography extracts information of the heterogeneity by fitting the observed waveforms with the simulated waveforms based on a physical principle (i.e., forward modeling), assuming that the source and the receiver locations are known. In this approach, the sensitivity (often represented by the gradient of the misfit^7^) is of fundamental importance to determine the resolution capability of this approach. The sensitivity of waveform tomography is defined by the change in the goodness-of-fit of the simulated waveforms with respect to the change in the assumed heterogeneity, which indicates spatial locations where the assumed heterogeneity needs to be updated during a nonlinear optimization^3,7,8^.

The conventional principle behind calculating the sensitivity involves one source point, one observation point, and the Huygens’ principle^3,7,8^: an incident wave at a source point causes a disturbance in the material, a secondary wavefield is generated at a heterogeneity, and the total wavefield (incident and scattered waves) is measured at an observation point. The sensitivity is then calculated using two Helmholtz equations: one in which the incident wave propagates forward in time from the source point, and one in which the scattered waves propagate backward in time from the observation point (adjoint). These two wavefields have identical arrival times at the location where scattered waves are generated (i.e., at the location of the heterogeneity). The sensitivity is calculated by analyzing the amount of correlation between the two wavefields^3,7,8^.

In various disciplines, owing to the reduced footprint of sensors and the increased computational resources, measurements and data processing based on many spatial observation points have become common. For example, waves are measured with spatially dense sampling, e.g., on the surface of the Earth^9,10^ or along boreholes^11,12^, at the surface of or inside the human body^13,14^, and in civil engineering structures^13,15^. These developments have led to spatially and temporally high-resolution imaging of materials across scales^9,12,16–18^.

Using the conventional principle, the sensitivity from multiple sets of source-observation points is obtained by simply summing up the sensitivity from every single set^3,7,8^, due to the linearity of the problem. Although this approach improves the sensitivity estimation, it does not fully exploit the interrelation among the scattered waves. Suppose waveforms due to a remote seismic source are measured at an array of receivers. As the conventional physical principle addresses material heterogeneities that are present along the wave propagation paths starting from a source and ending at an observation point, small material perturbation between the observation points does not appear in the sensitivity, unless the heterogeneities around the source points and those present along the source-observation paths are sufficiently known. This limits the resolution of the heterogeneity and its temporal changes between the observation points, especially when the installation of physical wave sources at the location of the observation points is not desirable or feasible. For example, in geoscientific applications, monitoring is necessary when injecting fluids into the subsurface using boreholes, e.g., when recycled water is injected and stored in an aquifer for water resource management^19,20^, or treated water is injected in order to produce energy in geothermal fields or to store carbon dioxide in the subsurface^21^. In all these applications, detecting subsurface changes due to the replacement of fluid and changes in the pore pressure is crucial^19,22,23^. For this purpose, monitoring using receivers (sensors) located in the boreholes is generally performed due to the sensitivity of downhole sensors to changes at the target depths^24–26^. However, generally speaking, seismic sources are located at the surface, because installing sources in a borehole is expensive and cumbersome, and therefore feasible only in large, well-funded projects. Also, it is impossible to place seismic sources in a borehole when the borehole is inaccessible due to injection operations^20^. When monitoring is performed using downhole receivers and surface sources, in addition to time-lapse changes in the target area, the data also contain the effect of time-lapse changes occurring around the source point, e.g., due to environmental effects (like rainfall), which can jeopardize the entire time-lapse monitoring efforts^25^.

Earlier studies on Green’s function retrieval^9,27–33^ (also known as seismic interferometry or time-reversal acoustics) have tackled a similar problem. In those studies, correlation or convolution of measured waveforms is used to estimate the Green’s function (impulse response) between the observation points^31^, which enables one to obtain the wavefield without placing a physical source at the location of the observation points. This concept has been found useful in a variety of disciplines, e.g., medical diagnostics^30^, seismology^9^, exploration geophysics^28^, and material testing^29^. One would apply the conventional physical principle to the retrieved Green’s functions in order to calculate the sensitivity of waveform tomography. However, as the estimated Green’s functions generally contain errors due to violation of a number of assumptions^31^, simply applying the conventional principle introduces errors in the sensitivity of waveform tomography.

In this article, we present a novel physical principle for calculating and localizing the sensitivity of waveform tomography without having to know the heterogeneities around the source points and those along the path from the source to the observation point. As in the conventional approach, the new principle is based on the correlation between forward and backward propagating waves. However, utilizing a boundary integral representation, here the observed waves and their interferences are exploited in correlating the wavefields, in order to take into account the information shared by multiple observation points (including all wave phenomena occurring along the path, e.g., transmission, scattering and dissipation). This leads to capturing small changes in the material properties, which remain otherwise hidden.

In the following discourse, we discuss the new physical principle and show the key concept behind calculating the wave sensitivity in a data-driven manner using multiple observation points and the boundary integral representation. We use the boundary integral representation in order to extrapolate the wavefield, without knowing the heterogeneity between the source and the receiver array. We then formulate the adjoint of the extrapolation process, which localizes the sensitivity. We first present the evidence that this representation works in real-world data of scattered waves observed in the field, and illustrate how the novel principle localizes the sensitivity. Successful localization of sensitivity and resolution of material heterogeneities are then verified on an experimental dataset. Next, we explore the potential use of this localized sensitivity to quantify temporal variations of acoustic properties in saturated rocks due to changes in pore-pressure associated with subsurface fluid flow. Finally, we discuss the possibilities for various observation geometries and applications. Note that in this study we assume 2D measurements where the source and the receiver are located in a 2D plane. Such measurements correspond to, e.g., surface-wave measurements using seismometer arrays^9^, seismic and electromagnetic wave experiments using boreholes^11,12,34^, non-destructive material testing using embedded sensors^15^, or ultrasonic/microwave medical imaging with an array of surface/embedded sensors on/in the human body^14,35,36^.

## A novel principle

### Wavefield extrapolation using the boundary integral representation: field data validation

In classical wave theory, the boundary integral representation of wavefields^37^ has been explored to relate the response at one place to that at other places. The boundary integral representation has been exploited in the past, for example, in modeling wavefield^38^ and in retrieving Green’s functions^31^. More recently, the boundary integral representation was utilized in waveform tomography in order to improve the computational efficiency^39^. A specific boundary integral representation can be formulated depending on the assumptions of medium properties in the Green’s function and the boundary conditions^40,41^. We use the boundary integral representation in order to extrapolate the wavefield (see “Boundary integral representation”). Here we illustrate how the extrapolation works on field data.

**Figure 1:**
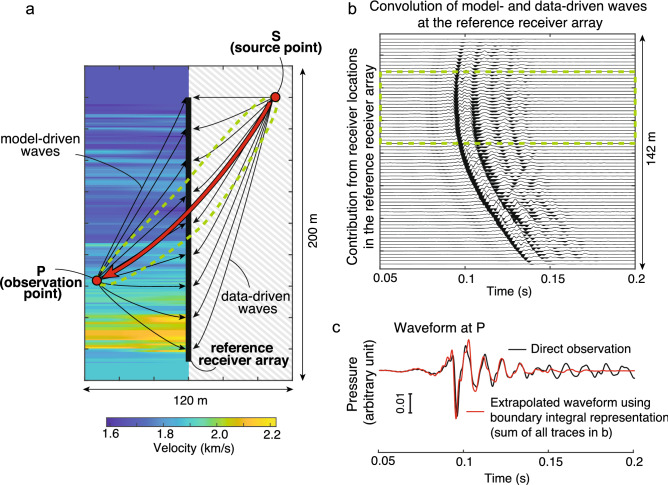
Wavefield extrapolation on actual field data using the boundary integral representation. Scattered waves are observed at a reference receiver array (pressure sensors). These waves are used in the representation to calculate the response at the observation point P (wavefield extrapolation). The black arrows schematically show wave propagation paths. (**a**) Source-observation point geometry and the velocity model assumed in calculating the “model-driven waves”. The process of wavefield extrapolation and the result do not need any knowledge of the velocity model in the gray-shaded area, and all wave phenomena (transmission, scattering, dissipation) in this zone are accounted for in a data-driven manner. The dashed line indicates a zone that contains wave paths that constructively interfere in the boundary integral representation. (**b**) Contribution at each receiver in the reference receiver array to the boundary integral representation. The dashed line shows a zone where waves constructively interfere. (**c**) The extrapolated waveform in comparison with the directly observed one at P.

As shown in Fig. 1a, suppose that the scattered waves are observed at multiple locations consisting of a single observation point P and an array of observation points marked as “reference receiver array”. Assuming a two-dimensional, scalar Helmholtz equation, we can adapt a boundary integral representation to calculate the response at P by extrapolating waves recorded at the reference receiver array. The vertical array for the reference receivers, as shown in Fig. 1a, corresponds to that located in a vertical borehole as we have used in the field experiment. However, this array can be arbitrary in shape. Let us consider an acoustic wavefield where the fluid pressure in a fluid-filled borehole due to an explosive seismic source located at the surface is measured. The measured data are preprocessed such that they represent the 2D wave propagation (see Supplementary Note 1). In this case, assuming that *N* receivers are located at a spacing of ∆*x* along the reference array (denoted as *x*_*i*_), the boundary integral representation can be written in the frequency domain as,

1 $$p(P,S) = \frac{ - 1}{{j\omega \rho }}\sum\limits_{i = 1}^{N} {\partial_{1} G(x_{i} ,P)p_{obs} (x_{i} ,S)\Delta x} ,$$

where *p*(*P*, *S*) is the extrapolated wave at P, *p*_*obs*_(*x*_*i*_, *S*) is the observed wave at the reference array, and ∂_1_*G* is the horizontal derivative of the Green’s function at the location of the vertical array (see “Boundary integral representation” for more details).

At first the impulse responses (“model-driven waves” in Fig. 1a), representing responses at the reference receiver array for an impulse at P (Green’s function *G*(*x*_*i*_, *P*) in Eq. 1), are calculated assuming an acoustic velocity structure which is independent of the heterogeneities present around the source point at S (gray-shaded area in Fig. 1a). This is because the model-driven waves or *G*(*x*_*i*_, *P*) do not radiate outward from the considered Dirichlet boundary (also commonly known as sound-soft or free-surface boundary) at the location of the reference receiver array. Then the boundary integral representation performs a temporal convolution between the model-driven waves and the observed waveforms (*p*_*obs*_(*x*_*i*_, *S*) in Eq. 1) at the reference receiver array (“data-driven waves” in Fig. 1a). This corresponds to the summation of traveltimes for travelpaths from S to the reference receiver array and that from P to the same array. The final response at P (“extrapolated waveform”) or *p*(*P*, *S*) is then obtained by collecting and adding contributions coming from all receivers in the reference array (Fig. 1b).

A part of the receivers (the area enclosed by the green dashed lines in Fig. 1a,b) in the reference array dominantly contributes to the final response due to the stationary phase, considering the Fresnel volume^42^. This indicates that all wave phenomena (transmission, scattering, and dissipation), which take place earlier than the time of arrival of the wave at the reference receiver array from the source S, are accounted for in a data-driven manner without a model. When we assume a realistic velocity model to calculate the model-driven waves, the extrapolated waveform matches excellently with the directly observed waveform (Fig. 1c). In the field data example shown in Fig. 1, the velocity model is derived from measurements of acoustic velocity at two vertical boreholes.

The result in Fig. 1c clearly shows that the boundary integral representation works very well on the real-world scattered wave data, and that it is possible to extrapolate responses at the observation point P without knowing the model around the source point S.

### The localized sensitivity using the adjoint of the boundary integral representation

Assuming negligible extrapolation error, any difference between the extrapolated and the directly observed waveforms is an indication that the assumed model deviates from the true model. Therefore, the sensitivity is defined as a change in the difference between the extrapolated and the observed waveforms due to a change in the assumed model^3,7,8^. In this study, we consider the difference between calculated and observed waveform defined as,

2 $$E(m) = \frac{1}{2}\sum\limits_{S,P,\omega } {\left\| {p - p_{obs} } \right\|^{2} } ,$$

where || ||^2^ denotes the squared L2 norm and the summation takes place over the number of frequencies, observation points, and source points, *p* and *p*_*obs*_ are respectively, calculated waveform using the boundary integral representation and observed waveform. The sensitivity is then defined as the multi-dimensional gradient of the misfit function (Eq. 2) with respect to the model (*m*), i.e., ∂*E*/∂*m*. This sensitivity can be utilized in waveform tomography (see “Waveform inversion”). In order to calculate the sensitivity, we adapt the adjoint-state method^7,43^ to our problem, i.e., minimizing the misfit function (Eq. 2) with the wave equation and the boundary integral representation (Eq. 1) as constraints. We first spatially discretize the boundary integral representation (Eq. 1) as,

3 $$p_{SP} = {\mathbf{p}}_{S} {\mathbf{g}}_{P} ,$$

where variables with subscript *S* indicate that they depend on the source location, those with subscript *P* indicate that they depend on the receiver location or the observation point P, and the frequency dependence of all variables is omitted for brevity. In Eq. (3), a column vector **g**_*P*_ is a solution to the wave equation^44^:

4 $${\mathbf{A}}(m){\mathbf{g}}_{P} = {\mathbf{f}}_{P} ,$$

where **A** represents the discretized Helmholtz operator which is a square matrix including the spatial distribution of material properties (acoustic velocity and density), finite-difference operator, and the Dirichlet boundary conditions at the location of the vertical array. A column vector **f**_*P*_ includes the discretized Dirac delta function at P. A row vector **p**_*S*_ in Eq. (3) approximates the spatial integral in Eq. (1): it includes the multiplication of data at the receiver array *p*(*x*, *S*) and scaling of –1/(*jωρ*). Here, **p**_*S*_ also contains the finite-difference operation (spatial differentiation) applied to the Green’s function in Eq. (1). In the case of the vertical array (Fig. 1), considering that the discretized Green’s function **g**_*P*_ is zero at the array due to the Dirichlet boundary conditions, **p**_*S*_ is the collection vector that collects and adds the values of **g**_*P*_ at one spatial grid on the left side of the array, followed by a scaling, as explained above.

The adjoint-state method provides an algorithm which calculates efficiently the gradient^7,43^. To this end, we define the Lagrangian of our problem (Eqs. 2, 3, and 4) as,

5 $$L\left( {\widetilde{p}_{SP} ,\widetilde{{\mathbf{g}}}_{P} ,\widetilde{\lambda }_{1} ,\widetilde{\lambda }_{2} ,m} \right) = \frac{1}{2}\sum\limits_{S,P,\omega } {\left\| {\widetilde{p}_{SP} - p_{SP}^{obs} } \right\|^{2} - \sum\limits_{S,P,\omega } {\widetilde{\lambda }_{1} \left( {\widetilde{p}_{SP} - {\mathbf{p}}_{S} \widetilde{{\mathbf{g}}}_{P} } \right) - \sum\limits_{S,P,\omega } {\widetilde{\lambda }_{2}^{T} \left( {{\mathbf{A}}(m)\widetilde{{\mathbf{g}}}_{P} - {\mathbf{f}}_{P} } \right)} ,} }$$

where the tilde “~” is introduced in order to distinguish between the physical realization and any element in the state and adjoint-state variable spaces, and $\widetilde{\lambda }_{1}$ and $\widetilde{\lambda }_{2}$ are the Lagrange multipliers. A saddle point of the Lagrangian provides the gradient of the misfit function represented by the adjoint-state variables^43^. The saddle point of the Lagrangian can be written as,

6 $$\frac{\partial }{{\partial m}}L\left( {p_{{SP}} ,{\mathbf{g}}_{P} ,\lambda _{1} ,\lambda _{2} ,m} \right) = \frac{{\partial E}}{{\partial m}} = - \sum\limits_{{S,P,\omega }} {\lambda _{2}^{T} \frac{{\partial {\mathbf{A}}}}{{\partial m}}{\mathbf{g}}_{P} .}$$

The real part is taken in the right-hand side term of the above equation because *E*(*m*) is real^43^. In the above equation, the adjoint-state variables (*λ*_1_, *λ*_2_) satisfy the following adjoint-state equations:

7 $$\lambda_{2}^{T} {\mathbf{A}}(m) = \lambda_{1} {\mathbf{p}}_{S} ,$$

8 $$\lambda_{1} = \left( {p_{SP} - p_{SP}^{obs} } \right)^{*} ,$$

where * denotes the complex conjugation. These equations provide an algorithm to calculate the localized sensitivity.

In order to interpret physically the adjoint-state equations, we rearrange the equations such that the sensitivity is the crosscorrelation of two wavefields **b** and **f**,

9 $$\frac{\partial E}{{\partial m}} = - \sum\limits_{S,P,\omega } {\Re [{\mathbf{b}}^{*}{\mathbf{f}}]} ,$$

where **f** is a column vector representing the scaled forward propagating wavefield **g**_*P*_ (Eq. 4) from the observation point P,

10 $${\mathbf{f}} = \frac{{\partial {\mathbf{A}}}}{{\partial m}}{\mathbf{g}}_{P} ,$$

where the term $\partial \mathbf{A}/\partial m$ compensates for the scattering radiation pattern due to different parameterization, and **b** is a row vector representing the backward propagating wavefield defined as,

11 $${\mathbf{A}}^{*}{\mathbf{b}}^T = \left( {p_{SP} - p_{SP}^{obs} } \right){\mathbf{p}}_{S}^{*T} .$$

The backward propagating wavefield **b** is a solution to the conjugate (time-reversed) wave equation where the source term represents the scattered waves (i.e., difference between calculated and observed waveforms) crosscorrelated with the observed waves at the reference receiver array. It is worth mentioning that the modeling operator **A** in Eq. (11) is the same as in Eq. (4) where the Dirichlet boundary condition is considered at the vertical array.

### Physical interpretation of the novel principle

**Figure 2:**
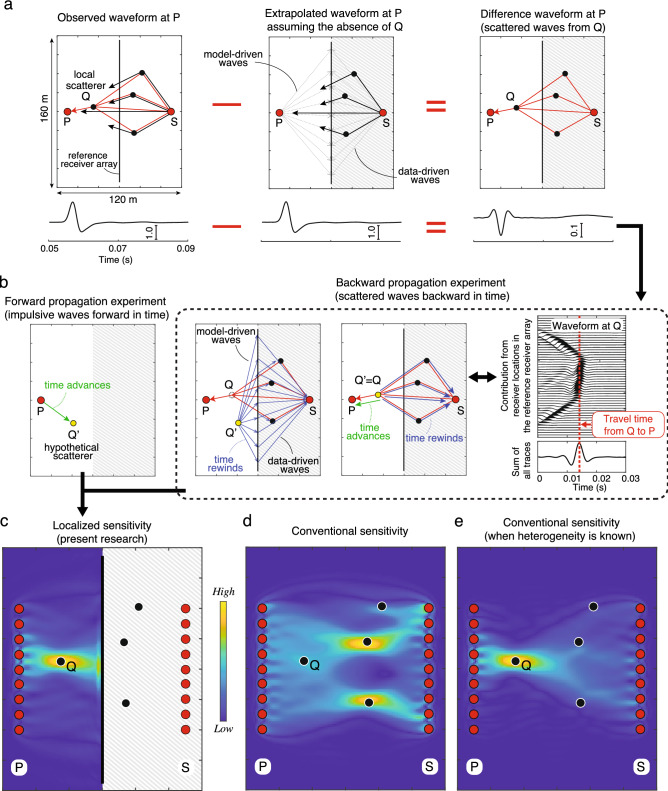
A novel principle to localize the sensitivity of waveform tomography: schematic illustration and results on synthetic data. (**a**) “Observed waveform”: all waves coming from a source S are observed at an observation point (P) and a reference receiver array. Black arrows show direct and scattered waves which are not associated with the local scatter Q located close to P, and red arrows show scatterings from Q. “Extrapolated waveform”: the waveform is extrapolated using the boundary integral representation assuming a material without Q. “Difference waveform”: the difference between the observed and the extrapolated waveforms containing scattered waves from Q. Gray-shaded area shows the zone which has no effect on the boundary integral representation. (**b**) Thought experiments of forward and backward wave propagation for calculating the localized sensitivity. The backward propagation experiment uses the difference waveform in (**a**) as input, and calculates the adjoint of the boundary integral representation (see main text). Both forward and backward propagation experiments are independent of the structure around the source point (gray-shaded area). (**c**) The localized sensitivity obtained from the correlation between the forward and the backward propagating waves for various locations of the hypothetical scatterer Q’. The sensitivity using multiple source-observation points is shown. (**d**) The conventional sensitivity obtained using the same source-observation points as in the calculation for the localized sensitivity. (**e**) The conventional sensitivity when all heterogeneities/scatterers but Q are known.

Let us consider a material which contains multiple scatterers (black circles in Fig. 2a). The measurement geometry is similar to Fig. 1a. Suppose we are interested in finding any heterogeneity between the reference receiver array and the observation point P where there is a local scatterer Q with a smaller scattering strength than the other scatterers. We first extrapolate the waveform at P (*p*_*SP*_ in Eq. 3) using the boundary integral representation along with the observed waves at the reference array and assuming homogeneity around P (the medium to the left of the vertical receiver array in Fig. 2a is assumed homogeneous, while the gray-shaded part on the right—located close to the source—does not need any such assumption). Note that arbitrary velocity structure can be assumed in the extrapolation, but here we take a homogeneous medium so that the calculated sensitivity shows a deviation from homogeneity. Since the boundary integral representation is independent of the knowledge of any heterogeneity around the source, any difference between the observed $\left( {p_{SP}^{obs} } \right)$ and the extrapolated (*p*_*SP*_) waveforms is caused by the disturbance in the wavefield due to the unknown heterogeneity close to the observation point P or due to the local scatterer Q (red arrows in Fig. 2a), and does not have any effect of the scattering (black arrows in Fig. 2a) that occurs around the source point. This feature, where the difference waveform contains the disturbance only due to the heterogeneity in the target zone between the reference array and P, improves the sensitivity to the local scatterer Q, without the need to know the position and/or the nature of the scatterers located between the source point and the reference receiver array (gray-shaded area in Fig. 2a).

Using the difference waveform, i.e., $p_{SP}^{obs} - p_{SP}$, we now introduce a novel physical principle based on the boundary integral representation. The application of the adjoint-state method^7,43^ to the discretized equations enables us to explain the principle using two wave-propagation thought experiments: forward (Eqs. 4 and 10) and backward (Eq. 11) propagations, as illustrated in Fig. 2b. In the forward propagation experiment, suppose that an impulsive wave propagates from the observation point P to a hypothetical scatterer Q’ (i.e., solving Eq. 4 for **g**_*P*_). The green arrow in Fig. 2b shows the wave propagating forward in time with a traveltime which is larger than zero. Next, in the backward propagation experiment, the difference waveform (red arrows in Fig. 2a) is crosscorrelated with the data-driven waves (observed waveforms at the reference receiver array), which corresponds to evaluating the right-hand side of Eq. (11). The waveform thus obtained is then back-propagated in time from the reference receiver array to the hypothetical scatterer Q’ (i.e., by solving Eq. 11 for **b**). This gives the model-driven and data-driven waves shown by the blues arrows in Fig. 2b. These arrows illustrate the above-described process, marking backward propagation in time for waves with traveltimes smaller than zero.

Crosscorrelation corresponds to subtraction of traveltimes; therefore the data-driven waves are represented by the blue arrows in Fig. 2b. Consequently, collecting all contributions from the reference receiver array and adding them result in the scattered waves (red arrows in Fig. 2b) which propagate backward from the hypothetical scatterer to the source point, leaving only the forward propagating wave travelling from Q to P (green arrow in Fig. 2b). In this way, by analyzing the amount of correlation between the forward and the backward propagating waves (Eq. 9), a large sensitivity can be achieved at the true scatterer Q in a data-driven manner, without having to know the heterogeneity around the source point.

This novel principle calculates the sensitivity which is highly localized at the location of the scatterer Q, without utilizing any knowledge of the complete heterogeneity distribution (Fig. 2c). In contrast, the sensitivity obtained from the conventional principle, using the same source-observation points, does not allow detecting the local scatterer Q (Fig. 2d). The same conclusion can be drawn even when we use in the calculation all available data (see Supplementary Fig. 1). When we assume that all heterogeneities but Q are perfectly known, the conventional sensitivity approaches the localized sensitivity that we estimate using the new principle (Fig. 2e). In other words, the conventional principle can address small heterogeneities only when the heterogeneities close to the source point are sufficiently known. On the other hand, the new principle, by exploiting the interference of the observed scattered waves and its adjoint, makes it possible to localize the sensitivity very accurately, as if we have a-priori the complete and accurate knowledge of all heterogeneities.

## Results of field test and value of the localized sensitivity

### Field test on sensitivity localization

**Figure 3:**
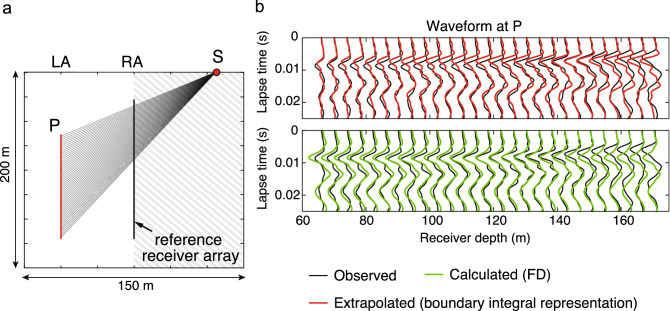
Observed and calculated/extrapolated waveforms in field measurements: (**a**) geometry of a surface source (S), an array of observation points (P), and a reference receiver array. Receivers are located in two vertical boreholes (LA, left array and RA, right array). (**b**) Observed waveform, extrapolated waveform using the boundary integral representation, and calculated waveform using conventional finite-difference method^44^. The time axis is the time lapsed since the expected arrival, assuming a constant acoustic velocity and straight propagation paths (black lines in (**a**)).

We have tested this new sensitivity localization principle on field experimental data. The measurement geometry is same as in Fig. 1a, but here we have multiple observation points P located along another vertical line (Fig. 3a): in total we observe wavefield using two vertical arrays (left array, LA, and right array, RA). In the field test, this corresponds to measurements in two vertical boreholes.

For the sake of clarity, here we need to distinguish between extrapolated and calculated waveforms. Extrapolated waveforms are obtained using the boundary integral representation that we have presented above and assuming homogeneity (constant acoustic velocity). The calculated waveform, on the other hand, are the ones obtained through numerical (finite-difference, FD) computation also assuming homogeneity, where the source wavelet is estimated by deconvolution of the recorded waveform^44^ with a waveform that is calculated assuming the same homogeneity and an impulsive source at S. Therefore, the difference in waveforms between the observation (black lines in Fig. 3b) and the calculation or extrapolation (green and red lines in Fig. 3b) indicates the deviation from the homogeneity. The waveforms calculated using the conventional approach by FD method (green lines in Fig. 3b) assume a globally homogeneous material. On the other hand, those using the boundary integral representation (red lines in Fig. 3b) assume local homogeneity between LA and RA, and heterogeneities around the source point (gray-shaded area in Fig. 3a) are accounted for in a data-driven manner. As a result, their waveforms vary over the receiver location (red lines in Fig. 3b), which implies an improved sensitivity to the local heterogeneity.

**Figure 4:**
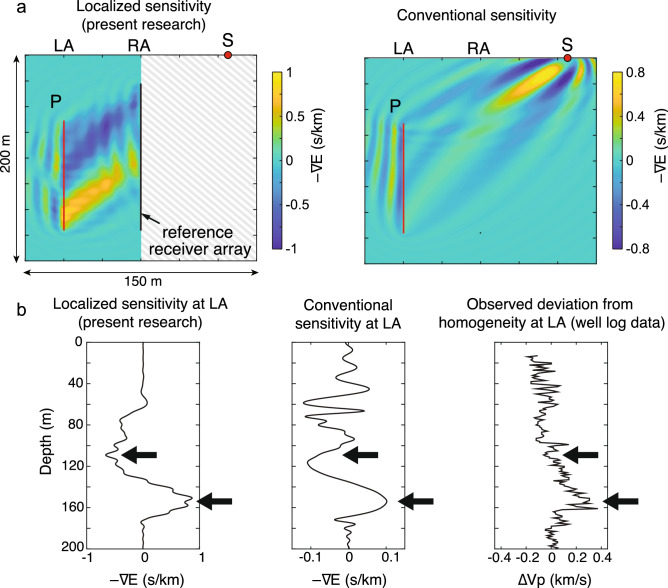
Localized sensitivity detected in field experimental data: (**a**) Localized and conventional sensitivity using the same source-observation points. (**b**) Comparison between the sensitivities and the observed perturbation (well log data) in acoustic velocity from a constant average value at LA. Black arrows mark the location where the localized sensitivity captures a finer variation in the structure than the conventional sensitivity.

The sensitivity localization is evident in Fig. 4a. The conventional sensitivity shows large values around the source point S. Further, the conventional sensitivity varies smoothly in the subsurface, which implies small correlation between the incident and the scattered waves (averaging out of the contribution of different local scatterers). This is due to the fact that in the conventional approach, the difference waveforms have a complex nature as they include more scatterers (see black and green lines in Fig. 3b). In contrast, the localized sensitivity, derived from the new principle found in this research, reveals a very detailed structure between LA and RA (Fig. 4a). The sensitivity indicates the amount of velocity perturbation/changes with respect to homogeneity, assuming Born scattering^45^. A comparison with the heterogeneity directly observed at LA (Fig. 4b) shows that the localized sensitivity detects a much finer variation in heterogeneity than the conventional sensitivity at depths greater than 80 m. The novel principle exploits information in the observed data in a completely different manner than the conventional principle. As a result, the conventional sensitivity cannot achieve comparably good results even using all available data, including data from the reference receiver array (Supplementary Fig. 2).

### Localized sensitivity: quantitative estimation of heterogeneity

The localized sensitivity can be exploited to resolve quantitatively the material heterogeneity. An inversion scheme can be formulated to estimate the acoustic velocity distribution by minimizing the difference between the observed and the calculated waveforms at the observation point. The localized sensitivity can navigate iteratively toward a best-fit model using nonlinear inversion (see “Waveform inversion”) without a knowledge of the heterogeneity around the source points.

**Figure 5:**
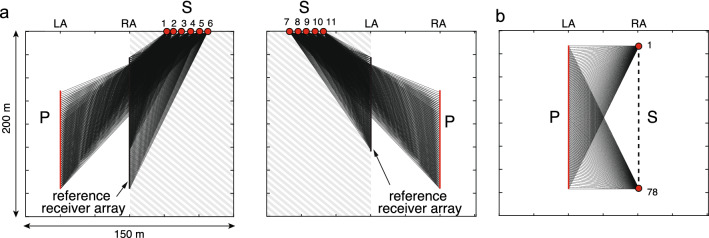
Schematic illustration of field experiments with multiple source-observation points: (**a**) multiple sources (S), an array of observation points (P), and the reference receiver array in the field experiments where surface sources are located on the right to RA or on the left to LA. The gray-shaded area marks the zone which has no effect on the calculated sensitivity (this research). (**b**) Ground-truthing measurements performed using downhole sources.

Using the same geometry as in Fig. 3a, multiple sources are used sequentially in the field to generate pressure waves at right to RA and left to LA (Fig. 5a) in order to illuminate the medium from various directions. The reference receiver array, the observation point (P), and the zone which does not contribute to calculating the localized sensitivity are appropriately defined depending on the source location (Fig. 5a). In order to verify the resolved heterogeneity, we additionally perform independent waveform measurements (ground-truthing) using downhole sources (Fig. 5b) and apply the conventional waveform inversion (Supplementary Note 2).

**Figure 6:**
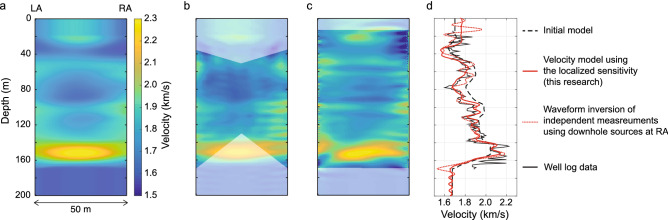
Heterogeneity resolved quantitatively on field data: (**a**) velocity structure derived from traveltime tomography, which is used as the initial model in waveform inversion. (**b**) Resolved structure using the localized sensitivity (this research). White-shaded area indicates a zone with low ray coverage. (**c**) Resolved structure from waveform inversion of data using downhole acoustic sources located at RA. (**d**) Comparison of obtained heterogeneous velocity distributions with well log data at RA.

Waveform inversion estimates a velocity model starting from an initial guess^3,7,8^. We perform standard traveltime tomography to obtain the starting model (Fig. 6a). Waveforms around the first-arriving events and a frequency component similar to that in the independent measurements using downhole sources are analyzed (Supplementary Notes 3 and 4). Figure 6b shows the estimated velocity structure using the localized sensitivity derived from the novel principle involving the boundary integral representation. Figure 6c shows the result of waveform inversion where additional downhole sources have been placed in RA (ground-truthing). Figure 6d shows in details a comparison between the different velocity models. The estimated velocity using the localized sensitivity is strikingly close to the one obtained from independent waveform inversion using downhole sources and also to acoustic well log data at RA, especially at depths greater than 80 m where the raypath-coverage is good (Fig. 6d).

### Value of the enhanced localization of sensitivity in geophysical monitoring

The novel principle presented in this article localizes the sensitivity without any knowledge of the heterogeneity around a source point. This has a significant merit in overcoming the inherent limitations of time-lapse data analyses, where the accuracy of temporal changes occurring in a target area is important, but is affected by structures located outside the target area. Past seismic experiments have demonstrated that ignoring errors in source location^46^ or near-surface changes due to seasonal variations in e.g., water saturation^25^ leads to spurious waveform changes and can jeopardize critical time-lapse seismic monitoring efforts. The use of the new principle enables overcoming these limitations by suppressing such spurious waveform changes (see Supplementary Note 5).

**Figure 7:**
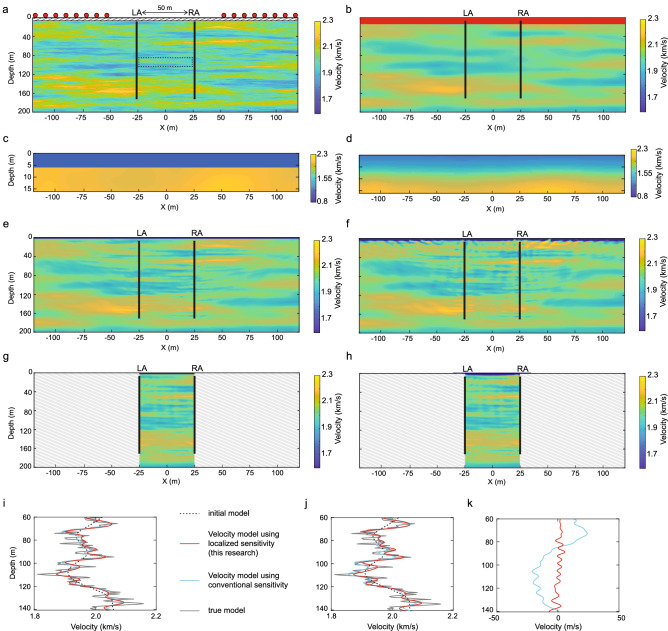
True and resolved velocity structures in the numerical monitoring experiments using waveform inversion with different accuracies in the prior knowledge of the vadose zone: (**a**) true velocity model (baseline). The topmost layer (hatched area) is the vadose zone. The dashed rectangle at 100 m depth indicates the target area where there are velocity changes between the baseline and the monitor surveys due to changes in the pore pressure. (**b**) Initial velocity model. (**c**) Initial velocity model for the top 16 m (red area in (**b**)); the correct thicknesses and the correct average velocity in the vadose zone are known. (**d**) Same as (**c**) but with poor prior information of the vadose zone. (**e**) Obtained result using conventional sensitivity when an accurate information of the vadose zone is available as in (**c**), and (**f**) the obtained result corresponding to (**d**). (**g**, **h**) Same as (**e**) and (**f**) but using localized sensitivity derived from the novel principle found in this research. (**i**) Comparison of the resolved velocity structures at the target area at x = 0 m using the prior information presented in (**c**), and (**j**) the same using prior information presented in (**d**). (**k**) Spurious velocity changes due to different accuracies in the prior knowledge of the vadose zone. The difference in the estimated velocity between (**i**) and (**j**) is shown using conventional sensitivity (blue line) and localized sensitivity (red line) for waveform tomography.

We delve further into this concept through performing realistic synthetic monitoring tests. As we explained in the introduction, this novel principle will be useful when having a physical source at the location of the observation points is not feasible. In our synthetic test we consider the borehole monitoring during fluid injection where the observation points are located in boreholes and the source points only at the surface (Fig. 7a), similar to field experiments discussed earlier in this article.

To generate realistic synthetic data, we assume a random velocity distribution with a mean velocity of 2.0 km/s (Fig. 7a). The data contain effects due to source location errors and those due to temporal changes occurring outside the target area (the dashed rectangle in Fig. 7a). The target area is located at 100 m depth where the velocity decreases by 5% with respect to the baseline measurement due to an increase in the pore pressure^47^. The topmost 6 m is modeled as a vadose zone having a random velocity distribution with a mean value of 1.0 km/s (Supplementary Fig. 7). Additionally, the structure of the vadose zone is completely different between the baseline and the monitor surveys (Supplementary Fig. 7), representing a possible drastic change in seismic velocity in this zone due to seasonal change in water saturation^25,48^. Source-receiver geometry and frequency components are quite similar to those in the field experiments discussed earlier (Supplementary Note 6). We consider a realistic scenario where the location of the receivers in the borehole is well calibrated and does not change during the time-lapse measurements (e.g., in the case of permanent monitoring), and the repeatability of the surface seismic sources (portable/mobile sources, e.g., vibrator) is limited due to, for instance, location errors. We assume that the source location in the monitor survey contains random errors up to 4 m (Supplementary Fig. 7).

We first look at the result of imaging the inter-borehole velocity heterogeneities in the baseline measurement. Here we explore two different scenarios for obtaining prior information regarding the non-target zone (outside the two boreholes) to build an initial velocity model for waveform inversion. Assuming the same initial velocity model for depths greater than 16 m (Fig. 7b), we consider the situation where the correct average velocity and thickness of the vadose zone are known (Fig. 7c), and in another case where we have a poor knowledge of them and only a smoothed velocity model is available (Fig. 7d). These two different initial velocity models are used to estimate the heterogeneities using the conventional sensitivity (Fig. 7e,f) and the localized sensitivity (Fig. 7g,h), respectively. As the recorded waveforms contain information of the structure present along the wave propagation path connecting the surface source and the downhole receiver, the waveform inversion using the conventional sensitivity estimates the heterogeneities not only in between the boreholes but also outside, i.e., those structures to the left of LA and to the right of RA (Fig. 7e,f)—which are not of interest. More critically, the estimation of the velocity structure in between the borehole has been influenced by the accuracy of prior information of the structures outside the two boreholes, contributing to large uncertainties in the estimated inter-borehole heterogeneities (Fig. 7i–k). On the contrary, the new principle presented in this research addresses the localized sensitivity and, therefore, provides directly the inter-borehole structure (Fig. 7g,h)—which is minimally influenced by the accuracy of the prior information of the non-target zone (Fig. 7k).

In order to achieve accurate results using the conventional sensitivity, it is crucial to account for the propagation effects outside the target zone (gray-shaded area in Fig. 7g,h) by obtaining independently good prior information. Alternatively, one can design carefully a multi-scale inversion scheme utilizing sequentially data from lower to higher frequencies in order to avoid gaps in the wavenumber information^3^. However, this is not a trivial task due to the difficulty in acquiring low-frequency data using controlled sources^3^ and because each frequency component has generally a different signal-to-noise ratio. The localized sensitivity is free from uncertainties associated with these fundamental limitations, because the propagation effects outside the target zone are accounted for in a data-driven manner (recall Fig. 2b,c where the propagation effects at the non-target area are taken into account without explicitly knowing the heterogeneity).

**Figure 8:**
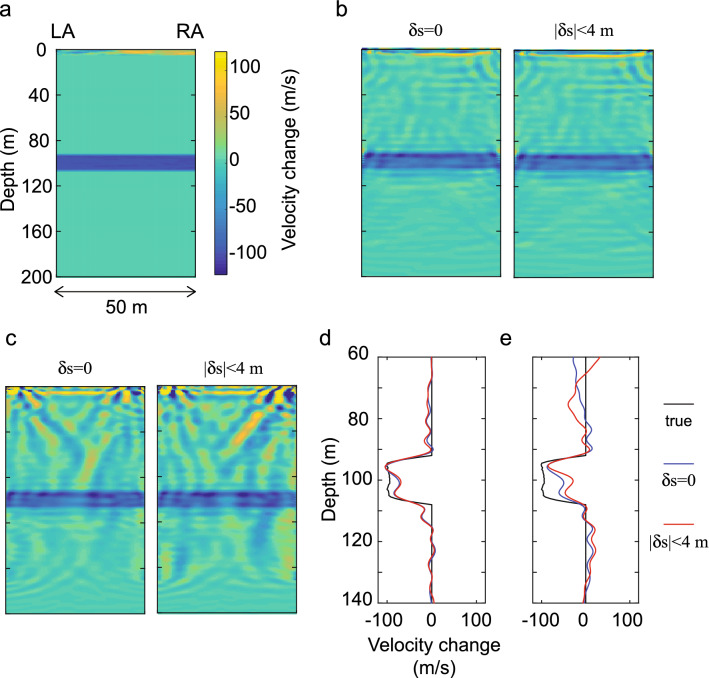
Time-lapse changes at the target zone (dashed rectangle in Fig. 7a): (**a**) true temporal changes. (**b**) Estimated temporal changes using the localized sensitivity for waveform tomography (this research). In this case, the initial velocity model contains inaccurate prior information regarding the vadose zone (Fig. 7d). Results using data without source location errors (δs = 0) and with source location errors (|δs| < 4 m) are shown. (**c**) Same as (**b**) but using the conventional sensitivity. (**d**) Estimated velocity changes in the target area at x = 0 m using the localized sensitivity, and (**e**) the same using the conventional sensitivity.

Next we concentrate on the monitoring of time-lapse changes in the target zone which is located around 100 m depth (dashed rectangle in Fig. 7a). The results are shown in Fig. 8. The new principle estimates the temporal changes at the target depth much better than the conventional approach (Fig. 8b,c). The conventional approach is sensitive to the source location errors in case an accurate, prior information of the vadose zone is available (Supplementary Fig. 8). Generally, the conventional approach requires an accurate prior knowledge of the vadose zone (Fig. 7). Any inaccuracy in this prior knowledge results in a significant loss of accuracy in the estimated time-lapse changes at the target zone in this case (Fig. 8c,e). On the contrary, the extremely high sensitivity of the new approach to the inter-well structures allows high-resolution estimation of the velocity changes, which is nearly independent of the presence of any source location error and/or inaccuracy in the prior information of the vadose zone (Fig. 8b,d, Supplementary Fig. 8).

## Discussion

In this article, we present a novel principle to localize the sensitivity of waveform tomography to medium heterogeneities, which is otherwise difficult to obtain in case of using the conventional principle for sensitivity estimation. As we explained in the introduction, earlier studies on Green’s function retrieval^9,27–33^ have tackled a similar problem from a different point of view. Although there is a good similarity between Green’s function retrieval and the concept presented here, there are also notable differences. The major difference is that our primary purpose is to directly obtain the sensitivity using the adjoint of the boundary integral representation and, therefore, the associated Green’s functions are by-products. This enables us to tackle the problem from a completely different point of view. We have used the Dirichlet boundary condition in the Green’s functions in a rather unconventional manner (see “Boundary integral representation”). This makes it possible to relax the critical assumption of one-way wavefield propagation in the Green’s function retrieval using convolution^32,33^ and that of multi-component measurements or single-component measurements with approximations^31,41^. The assumption of one-way wavefield and/or the approximations due to the conventional boundary condition are otherwise necessary when the primary purpose is to retrieve Green’s functions, which will require further processing.

We have formulated the new principle as a 2D problem. We have shown in this article that the assumption of 2D wave propagation is effective for field data. Also, the geometry of the reference receiver array and the observation point can be arbitrary. In this regard, a similar concept, but using a conventional integral representation for Green’s function retrieval, was proposed earlier for reflected waves where the reference receiver array and the observation points are co-located in a horizontal borehole^49^. Also, this newly found principle can be applied to seismological monitoring using surface-waves and 2D seismometer arrays because single-mode surface waves in 3D elastic media can be represented by 2D wave propagation at the surface^50^. The independence of the estimated localized sensitivity from source locations and heterogeneities around the source points is attractive for ambient noise tomography, where the limitation due to uneven distribution of noise sources and due to heterogeneities outside the target area is especially detrimental to imaging and monitoring^51^. The novel principle can also be extended to 2D P-SV and 3D wave propagation problems. In the former, one replaces the acoustic boundary integral representation by that of an elastic field^40^, which will require vector-field measurements (e.g., vertical and horizontal displacements) at a reference receiver array. In the latter case, one needs to measure waves at the reference array located over a 2D surface.

The novel principle provides a unique opportunity in case the wave source does not illuminate a medium from a location which is close to the target area, but multiple observation points are used to enhance the localization of the sensitivity without the need to know precisely the structures outside the target area. We have illustrated that this principle is especially useful in monitoring, where the subsurface is illuminated by distant sources and the response is observed by embedded sensors. In other disciplines, this may necessitate a new data-acquisition design. In this regard, the development of fiber optic sensing has lately demonstrated that existing telecommunication networks can turn into spatially dense, subsurface (i.e., pseudo-borehole) acoustic receivers without a need of additional sensor installation^10,52^. This can be exploited to resolve/monitor a structure between the subsurface fibre cables only using remote seismic sources (e.g., those at the surface). The novel principle can, therefore, be powerful in future seismic monitoring in areas with difficult access, e.g., in urban or underwater environments. We anticipate that this principle will open up possibilities for new experiments and measurement techniques where accurate and efficient monitoring is of high importance but the conventional approaches using scattered waves are hindered by the insensitivity to the target area due to limitations in data-acquisition geometry or a poor knowledge about changes occurring outside the target zone.

## Methods

### Boundary integral representation

The following boundary integral representation is used to calculate the waveform at the observation point at P due to the source point at S using interferences of the observed waveforms at the reference receiver array:

12 $$p(P,S) = \frac{ - 1}{{j\omega \rho }}\oint\limits_{\partial D} {\sum\limits_{i = 1}^{2} {n_{i} \partial_{i} G(x,P)p(x,S)\;ds} ,}$$

where all properties are in the space-frequency domain, *ω* is the angular frequency, *j* the imaginary unit, *ρ* the density, *ds* the line element, and *n*_*i*_ the outward pointing normal vector on the arbitrary integral path ∂*D,* which is the location of a reference receiver array. Note that in actual data processing, we obtain frequency-domain signals by Fourier-transforming time-domain signals with an additional damping function (Supplementary Notes 3 and 4). Equation (12) indicates that the multiplication of the observed waveform, *p*(*x*, *S*) where *x* ∈ ∂*D*, at the receiver in the reference array and the spatial derivative of the Green’s function, ∂_*i*_*G*(*x*, *P*), in the *i* direction due to a point source at P, and the collection of its contributions from all receivers calculate the observed waveform at P*.* We derive Eq. (12) from the general wavefield representation^40^ where arbitrary velocity structure and boundary conditions can be assigned for the Green’s function. Here, we define the Green’s function such that the velocity structure is same as that of the observed data, but with the Dirichlet (sound-soft) boundary condition at ∂*D*. This additional boundary condition correctly handles the outward propagating waves at the reference receiver array by canceling non-physical wave arrivals while evaluating the integral. Furthermore, it enables us to require only single component wavefield measurements (e.g., pressure field instead of pressure and particle velocity fields), and the approximations due to single component measurements^31,41^ are not necessary. This contrasts with other similar techniques of wavefield retrieval^32,33^. Furthermore, the boundary condition enables us to use model information only inside the reference receiver array because waves in the impulse responses (Green’s function) do not radiate outward from the boundary.

The array shape (∂*D*) in Eq. (12) is arbitrary. We consider the special case of a vertical line (Fig. 1a). Suppose that a source S is located to the right of the reference receiver array, and the observation point P is located to the left of the reference receiver array. In this configuration, Eq. (12) can be written as

13 $$p(P,S) = \frac{ - 1}{{j\omega \rho }}\int {\partial_{1} G(x,P)p_{obs} (x,S)ds} ,$$

where *p*_*obs*_ is the observed waveform at the reference receiver array, *G* is the Green’s function with the Dirichlet boundary condition at the horizontal location of the reference receiver array (Fig. 1a), and we use the relation (*n*_1_, *n*_2_) = (1, 0). Equation (13) can be derived from Eq. (12) using the same approach as that well known in the study of the Green’s function retrieval^53^ where the closed contour ∂*D* is divided into the two parts (a vertical line and a half-circle), and we let the radius of the half-circle go to infinity, which leaves only the integral along the vertical line. Equation (13) is accurate, i.e., no approximation is involved except for the constant density along ∂*D*, as long as ∂*D* is infinitely long and located between P and S. In a practical situation where the vertical line is finite, Eq. (13) is useful to represent waves that travel from S to P passing through the vertical line. More details of the use of the finite array can be found in the studies of the Green’s function retrieval using boreholes^32,33^.

### Field experiment

The test site is made of sedimentary layers. Two instrumented vertical boreholes with 50 m horizontal separation are available. Hydrophone strings, installed in 28–170 m depth range with 2 m separation between two adjacent hydrophones, are used to measure the pressure wavefield due to a surface source, simultaneously in the two boreholes. We use a small amount (6 g) of explosives as surface sources. The measurement-depth interval is split into four sections. The receiver arrays (hydrophone strings) are positioned simultaneously at one of the sections in each borehole; they measure the pressure wavefield due to the surface source. In order to cover the measurement-depth interval, we repeat this procedure four times at the fixed source location changing the depth of the receiver arrays. The total record length is 0.4 s with a sampling interval of 0.25 ms.

### Waveform inversion

We use the quasi-Newton *l*-BFGS method^54,55^ in estimating the velocity model through waveform inversion. The model parameter is iteratively updated using the following formula:

14 $$m_{{k + 1}} = m_{k} - \alpha _{k} Q_{k} \frac{{\partial E(m_{k} )}}{{\partial m}},$$

where *Q*_*k*_ is the approximate Hessian inverse computed using previous values of the gradient, and *α*_*k*_ is the step length in the line-search in the descent direction.

## Supplementary Information

Supplementary Information.

## Data Availability

The datasets generated and/or analysed in the current study are available from the corresponding author on reasonable request.

## References

[1] Nikolova NK (2011). Microwave imaging for breast cancer. IEEE Microw. Mag..

[2] Wiskin J, Borup DT, Johnson SA, Berggren M (2012). Non-linear inverse scattering: High resolution quantitative breast tissue tomography. J. Acoust. Soc. Am..

[3] Virieux J, Operto S (2009). An overview of full-waveform inversion in exploration geophysics. Geophysics.

[4] Fichtner A, Kennett BLN, Igel H, Bunge HP (2008). Theoretical background for continental-and global-scale full-waveform inversion in the time–frequency domain. Geophys. J. Int..

[5] Klotzsche A, van der Kruk J, Linde N, Doetsch J, Vereecken H (2013). 3-D characterization of high-permeability zones in a gravel aquifer using 2-D crosshole GPR full-waveform inversion and waveguide detection. Geophys. J. Int..

[6] Wai-Lok Lai W, Dérobert X, Annan P (2018). A review of Ground Penetrating Radar application in civil engineering: A 30-year journey from Locating and Testing to Imaging and Diagnosis. NDT E Int..

[7] Tromp J (2020). Seismic wavefield imaging of Earth’s interior across scales. Nat. Rev. Earth Environ..

[8] Tarantola A (1984). Inversion of seismic reflection data in the acoustic approximation. Geophysics.

[9] Shapiro NM, Campillo M, Stehly L, Ritzwoller MH (2005). High-resolution surface-wave tomography from ambient seismic noise. Science.

[10] Lindsey NJ, Craig Dawe T, Ajo-Franklin JB (2019). Illuminating seafloor faults and ocean dynamics with dark fiber distributed acoustic sensing. Science.

[11] Daley TM (2013). Field testing of fiber-optic distributed acoustic sensing (DAS) for subsurface seismic monitoring. Lead. Edge.

[12] Binley A, Winship P, Middleton R, Pokar M, West J (2001). High-resolution characterization of vadose zone dynamics using cross-borehole radar. Water Resour. Res..

[13] Drinkwater BW, Wilcox PD (2006). Ultrasonic arrays for non-destructive evaluation: A review. NDT E Int..

[14] Poeggel S (2015). Optical fibre pressure sensors in medical applications. Sensors (Switzerland).

[15] Lynch JP, Wang Y, Loh KJ, Yi JH, Yun CB (2006). Performance monitoring of the Geumdang Bridge using a dense network of high-resolution wireless sensors. Smart Mater. Struct..

[16] Gruber FK, Marengo EA, Devaney AJ (2004). Time-reversal imaging with multiple signal classification considering multiple scattering between the targets. J. Acoust. Soc. Am..

[17] Gemmeke H, Ruiter NV (2007). 3D ultrasound computer tomography for medical imaging. Nucl. Instrum. Methods Phys. Res. A..

[18] Rabut C (2019). 4D functional ultrasound imaging of whole-brain activity in rodents. Nat. Methods.

[19] Sheng Z (2005). An aquifer storage and recovery system with reclaimed wastewater to preserve native groundwater resources in El Paso, Texas. J. Environ. Manage..

[20] Almalki M, Harris B, Dupuis JC (2013). Field and synthetic experiments for virtual source crosswell tomography in vertical wells: Perth Basin, Western Australia. J. Appl. Geophys..

[21] Evans KF, Zappone A, Kraft T, Deichmann N, Moia F (2012). A survey of the induced seismic responses to fluid injection in geothermal and CO_2_ reservoirs in Europe. Geothermics.

[22] Lumley DE (2001). Time-lapse seismic reservoir monitoring. Geophysics.

[23] Arts R (2003). Monitoring of CO_2_ injected at Sleipner using time lapse seismic data. Greenh. Gas Control Technol. 6th Int. Conf..

[24] Daley TM, Myer LR, Peterson JE, Majer EL, Hoversten GM (2008). Time-lapse crosswell seismic and VSP monitoring of injected CO_2_ in a brine aquifer. Environ. Geol..

[25] Ikuta R, Yamaoka K, Miyakawa K, Kunitomo T, Kumazawa M (2002). Continuous monitoring of propagation velocity of seismic wave using ACROSS. Geophys. Res. Lett..

[26] Mateeva A (2014). Distributed acoustic sensing for reservoir monitoring with vertical seismic profiling. Geophys. Prospect..

[27] Lobkis OI, Weaver RL (2001). On the emergence of the Green’s function in the correlations of a diffuse field. J. Acoust. Soc. Am..

[28] Snieder R, Miyazawa M, Slob E, Vasconcelos I, Wapenaar K (2009). A comparison of strategies for seismic interferometry. Surv. Geophys..

[29] Michaels JE, Michaels TE (2005). Detection of structural damage from the local temporal coherence of diffuse ultrasonic signals. IEEE Trans. Ultrason. Ferroelectr. Freq. Control.

[30] Sabra KG, Conti S, Roux P, Kuperman WA (2007). Passive in vivo elastography from skeletal muscle noise. Appl. Phys. Lett..

[31] Wapenaar K (2011). Seismic interferometry by crosscorrelation and by multidimensional deconvolution: A systematic comparison. Geophys. J. Int..

[32] van der Neut J, Thorbecke J, Mehta K, Slob E, Wapenaar K (2011). Controlled-source interferometric redatuming by crosscorrelation and multidimensional deconvolution in elastic media. Geophysics.

[33] Minato S (2011). Seismic interferometry using multidimensional deconvolution and crosscorrelation for crosswell seismic reflection data without borehole sources. Geophysics.

[34] Slob E, Sato M, Olhoeft G (2010). Surface and borehole ground-penetrating-radar developments. Geophysics.

[35] Meaney PM, Paulsen KD, Chang JT, Fanning MW, Hartov A (1999). Nonactive antenna compensation for fixed-array microwave imaging: Part II—Imaging results. IEEE Trans. Med. Imaging.

[36] Garcia-Pardo C (2018). Ultrawideband technology for medical in-body sensor networks: An overview of the human body as a propagation medium, phantoms, and approaches for propagation analysis. IEEE Antennas Propag. Mag..

[37] Morse PM, Feshbach H (1954). Methods of theoretical physics. Am. J. Phys..

[38] Fokkema JT, van den Berg PM (1993). Seismic Applications of Acoustic Reciprocity.

[39] Willemsen B, Malcolm A, Lewis W (2016). A numerically exact local solver applied to salt boundary inversion in seismic full-waveform inversion. Geophys. J. Int..

[40] Wapenaar K (2007). General representations for wavefield modeling and inversion in geophysics. Geophysics.

[41] Ramirez AC, Weglein AB (2009). Green’s theorem as a comprehensive framework for data reconstruction, regularization, wavefield separation, seismic interferometry, and wavelet estimation: A tutorial. Geophysics.

[42] Snieder R (2004). Extracting the Green’s function from the correlation of coda waves: A derivation based on stationary phase. Phys. Rev. E.

[43] Plessix RE (2006). A review of the adjoint-state method for computing the gradient of a functional with geophysical applications. Geophys. J. Int..

[44] Pratt G, Shin C, Hicks GJ (1998). Gauss-Newton and full Newton methods in frequency-space seismic waveform inversion. Geophys. J. Int..

[45] Sirgue L, Pratt RG (2004). Efficient waveform inversion and imaging: A strategy for selecting temporal frequencies. Geophysics.

[46] Landrø M (1999). Repeatability issues of 3-D VSP data. Geophysics.

[47] Christensen NI, Wang HF (1985). The Influence of pore pressure and confining pressure on dynamic elastic properties of Berea sandstone. Geophysics.

[48] Lu Z, Sabatier JM (2009). Effects of soil water potential and moisture content on sound speed. Soil Sci. Soc. Am. J..

[49] da Costa CAN, Costa JC, Medeiros WE, Verschuur DJ, Soni AK (2018). Target-level waveform inversion: A prospective application of the convolution-type representation for the acoustic wavefield. Geophys. Prospect..

[50] Tanimoto T (1990). Modelling curved surface wave paths: Membrane surface wave synthetics. Geophys. J. Int..

[51] Sager K, Ermert L, Boehm C, Fichtner A (2018). Towards full waveform ambient noise inversion. Geophys. J. Int..

[52] Ajo-Franklin JB (2019). Distributed acoustic sensing using dark fiber for near-surface characterization and broadband seismic event detection. Sci. Rep..

[53] Wapenaar K, van der Neut J (2010). A representation for Green’s function retrieval by multidimensional deconvolution. J. Acoust. Soc. Am..

[54] Nocedal J (1980). Updating quasi-Newton matrices with limited storage. Math. Comput..

[55] Métivier L, Brossier R (2016). The SEISCOPE optimization toolbox: A large-scale nonlinear optimization library based on reverse communication. Geophysics.

