# Supplementary material for: A novel principle to localize the sensitivity of waveform tomography using wave interferences at the observation boundary
Source: Sci Rep. 2021 Nov 11;11:22073. doi: 10.1038/s41598-021-01199-1 (PMC8586361; doi:10.1038/s41598-021-01199-1)
Supplement: Supplementary file 1 — Supplementary Information. [file 41598_2021_1199_MOESM1_ESM.pdf]

Supplementary Information for:

A novel principle to localize the sensitivity of waveform tomography using wave interferences at the observation boundary

Shohei Minato[1]*, Ranajit Ghose[1]

[1]Dept. of Geoscience and Engineering, Delft University of Technology, Stevinweg 1, 2628 CN Delft, Netherlands.
*Correspondence to: s.minato-1@tudelft.nl

Supplementary Note 1

The scattered waves recorded in the field data are preprocessed so that they represent two-dimensionally propagating waves, and that the data are simultaneously measured at all observation points. We first check visually the amplitude saturation and the noise interferences in the first arriving waves. We observe cable-traveling waves with lower velocity than the P waves; the traces in which these waves interfere with the first-arriving waves are excluded. We then pick the traveltimes of the first-arriving waves, and estimate the one-dimensional velocity distribution by fitting the observed traveltimes using a least-squares approach. Synthetic waveforms are calculated using the 1D velocity model and the finite-difference method. We compare amplitudes of the synthetic waveforms and those of the field waveforms in common shot gathers (CSGs). The root-mean-square (RMS) amplitudes of waveforms around the first-arriving waves are calculated, and their variation along receivers in the CSGs is compared between the synthetic data and the field data. Because of the repeatability issue of the surface sources, we find that, in several CSGs, the RMS amplitudes show discontinuity at boundaries between the sections in the measured depth interval. We compensate these amplitude discontinuities by scaling the data. The same scaling factor is applied to traces which belong to the same section at each borehole, considering the fact that the hydrophone arrays measure the wavefield simultaneously. The scaling factors are calculated from the synthetic waveforms such that they have the same average relative RMS amplitude at the two consecutive sections. We then apply running-average scaling (window length of three traces) calculated by the relative RMS amplitudes in order to smooth the variation of the amplitude along the receivers[1]. We mute the amplitudes recorded earlier than the first-arriving waves and those later than the shear-wave arrivals. Furthermore, in order to mitigate the effects due to variations of the wavelet within CSGs, we apply Wiener shaping filter. The input and reference wavelets for the shaping filter are calculated by averaging the time-gated first-arriving waves. Finally, the amplitude is scaled by $t^{1/2}$ in order to account for a difference between 2D and 3D geometrical spreading.

Supplementary Note 2

Independent waveform measurements (ground-truthing) are performed using physical downhole sources and geophones. The deployed downhole source generates a pulse in the horizontal direction in the borehole wall by mechanically compressing the borehole fluid[2,3]. We measure the vertical component of the particle velocity. The total recording length is 0.25 s with a sampling interval of 0.0625 ms. The measurement-depth interval is between 16 m and 170 m depth at 2 m spacing. The downhole receiver array, which consists of four geophones, is mechanically clamped to the borehole wall. The downhole source generates the seismic wave at each depth while the position of the receiver array is fixed. This procedure was repeated until we obtained all source-receiver pair data.

We pick the traveltimes of the first-arriving waves, and estimate the 2D velocity model. We use the least-squares approach with regularization[4], where an initial velocity model is calculated using the Back Projection Technique[5]. The estimated velocity model is shown in Fig. 6a. Next, we check the fidelity of the amplitudes in the data. To this end, we calculate the RMS amplitudes

of time-gated first-arriving waves. We then check the difference of the RMS amplitudes along source-receiver offsets from the one calculated from the synthetic data. The synthetic data is obtained using a finite-difference method that uses the tomographic velocity model (Fig. 6a). We repeat this procedure using common shot gathers (CSGs) and common receiver gathers (CRGs). We find that the variation of the RMS amplitudes along source-receiver offset is smoother in CRGs than in CSGs. This suggests that the repeatability of the downhole source is high and/or the impulse response at the receiver location is not homogeneous along the measurement depth. The latter is due to, e.g., variations in receiver-borehole wall coupling and variations in the sensitivity of geophones in the receiver array.

We estimate the source radiation pattern. To this end, the RMS amplitudes of the time-gated first-arriving waves are calculated after compensating for the effects of geometrical spreading. The effect of wave transmission across the impedance-contrast boundaries is ignored. The angle of propagation was calculated assuming straight travelpath between a source and a receiver. We compute the theoretical radiation pattern (vertical component) assuming a horizontal point force (blue line in the Supplementary Fig. 3). We confirm that the horizontal point force represents well, in the vertical plane, the radiation pattern of the theoretical borehole-source with radial stress applied to the borehole wall[6], assuming the seismic velocities of the elastic body surrounding the water-filled borehole to be same as those derived from the average values in the sonic log data. The estimated source radiation pattern (Supplementary Fig. 3) shows that the downgoing waves have larger energy than the upgoing waves. This could be explained as the energy produced by the downhole source decreases with depth and/or with the mechanical characteristics of the multi-disc structure of the downhole source, which could cause a lag time in the generating pulses at each discs[3].

The conventional waveform inversion is applied to the preprocessed downhole source data. The conventional waveform inversion minimizes the misfit function by solving the wave equation and modelling the downhole sources and receivers[7,8]. We use acoustic wave equation in the frequency domain. The conventional waveform inversion requires estimating the source term in the wave equation[7]. Because our data show excellent source repeatability, we use CRGs as input data. Consequently, we consider source-receiver reciprocity[9], and we solve the wave equation due to a vertical point force at the location of the receiver and calculate the horizontal particle velocity at the location of downhole sources. The source term in this case represents an effective source wavelet including geophone sensitivity and receiver-borehole wall coupling effect. The source term is estimated iteratively in the nonlinear iteration[7]. In order to take into account the strong energy difference between upgoing and downgoing waves (Supplementary Fig. 3), we assume that upgoing and downgoing waves are independent in the CRGs, and we can estimate individually the effective source wavelets.

The conventional waveform inversion is applied using the frequency component of 120 Hz and the initial velocity model (Fig. 6a). We apply time-shifted Laplace damping[10,11] with a damping factor of 100. After inversion using the *l*-BFGS method with the memory variable of 15 and 50 nonlinear iterations, the misfit function decreases to 5.1 % of the initial value. The final velocity model is shown in Fig. 6c. The observed data are calculated using this final velocity model, which fit the data much better than those derived from the initial model (Supplementary Fig. 4).

Supplementary Note 3

In order to analyze the waveforms around the early-arriving events, we apply the time-shifted Laplace damping[10,11] to the observed data. The damping function can be written as $exp(-\sigma t+\sigma T_0(P,S))$ where σ is the Laplace constant, and $T_0(P,S)$ is the traveltime of the first-arriving wave. Consequently, we modify the boundary integral representation to account for the damping function. The multiplication of $exp(-\sigma t)$ in the time domain corresponds to replacing angular frequency ω to be complex value, $\Omega = \omega - j\sigma$, in the frequency domain[10]. It is, therefore, straightforward to implement the time-shifted Laplace damping to the boundary integral representation:

$$p(P,S,\Omega)e^{\sigma T_0(P,S)} = \frac{-1}{j\Omega\rho}\oint_{\partial D}\sum_{i=1}^{2} n_i \partial_i G(x,P,\Omega) p(x,S,\Omega) e^{\sigma T_0(P,S)}\, ds. \qquad (1)$$

The left-hand side of above equation corresponds to the Fourier-transformed waveform data after applying the damping function. The gradient of the misfit function based on the above equation can be calculated in the same manner as the one without the Laplace damping, but replacing the frequency with a complex-valued one (Ω).

Supplementary Note 4

We apply the time-shifted Laplace damping (Supplementary Note 3) with a Laplace constant of 30. After Fourier transformation, we use the data at 125 Hz for the inversion. For the purpose of comparison with the calibration measurements using the physical downhole sources, we use the same initial velocity model as in the calibration measurements. The gradients are calculated separately using the data due to the sources to the left of LA and those to the right of RA, and the total gradient is calculated by summation. After inversion using the *l*-BFGS method with the memory variable of five and ten nonlinear iterations, the misfit function decreases to 9.0 % of the initial value. The observed data are calculated using the final velocity model, which predict the field-observed data much better than data obtained using the initial model (Supplementary Fig. 5).

Supplementary Note 5

In a numerical experiment (Supplementary Fig. 6a), the waveform recorded at an observation point P due to a point source at S, in a two-dimensional acoustic medium with random distribution of sound velocity, is computed using the FD method. We consider this as the observed data. In resolving the structure and the temporal changes occurring within that structure, one minimizes the difference between the observed waveforms and the waveforms calculated based on the known source and receiver positions and the velocity model. In

calculating the waveform, however, significant errors occur when the input source location is erroneous (Supplementary Fig. 6b) and/or when the heterogeneities outside the target area is ignored (Supplementary Fig. 6c). These errors lead to the estimation of spurious temporal changes in the target area. Using the boundary integral representation and a reference receiver array surrounding a target area, the observed waveform can be calculated without knowing the model outside the target area. The calculated waveform without any knowledge of the source location or of the structure outside the target area is nearly identical to the observed waveform (Supplementary Fig. 6d).

Supplementary Note 6

Time-lapse data are calculated using a finite-difference method. The random velocity perturbation in the baseline model is computed assuming the von Karman autocorrelation function[12] with a horizontal correlation length of 50 m, a vertical correlation length of 7 m, the Hurst number of 0.5, and an amplitude of 80 m/s. The top six meters are considered to be a vadose zone, which is modelled as a random-velocity medium with a slower background velocity of 1.0 km/s. At the vadose zone, a von Karman autocorrelation function is considered with a horizontal correlation length of 30 m, a vertical correlation length of 4 m, the Hurst number of 0.5, and an amplitude of 40 m/s. Different realizations of the random velocity models are assigned at the vadose zone between the baseline and the monitor surveys.

Two vertical boreholes with 50 m horizontal separation are considered. Receivers are installed at the boreholes between 2 m to 170 m depth at 2 m spacing, and 10 surface sources to the left of LA are regularly spaced between 5 m to 95 m distance from the LA. The sources to the right of RA are installed symmetrically with respect to those in the left. The locations of the surface sources in the monitor survey are assumed to have calibration errors; in calculating synthetic monitor data, the sources are not installed exactly at the desired locations. The source time function is a Ricker wavelet with centre frequency of 180 Hz.

The initial velocity model is constructed by smoothing the true baseline model using a Gaussian filter. We use the same initial velocity model for the baseline data and the monitor data. In both the proposed and the conventional approaches, we use as input data the frequency-domain waveform at five frequencies between 100 Hz and 200 Hz with a spacing of 25 Hz. The Laplace constant of 100 is used. In the proposed approach using the localized sensitivity, we apply *l*-BFGS method with a memory variable of 5; we perform 30 nonlinear iterations. When using conventional sensitivity, we apply *l*-BFGS method with a memory variable of 5, and we perform 40 nonlinear iterations. We repeat the procedure using the monitor data in order to estimate the temporal changes. Note that the conventional waveform inversion requires the source position to be known; we use the information of the source locations of the baseline measurements in processing both baseline and monitor data.

Assuming that we have a good prior information of the vadose zone and that the source location does not fluctuate between the baseline and the monitor surveys, both approaches estimate almost equally well the time-lapse velocity changes ($\delta s=0$ in Supplementary Fig. 8). However,

when the source location changes during the monitoring and we do not have correct information of source location/geometry, then spurious velocity changes appear in the conventional approach, which damages the imaging accuracy. The new approach presented here, however, does not require source location/geometry as input parameter in the inversion, and shows excellent robustness against possible error in source locations (Supplementary Fig. 8).

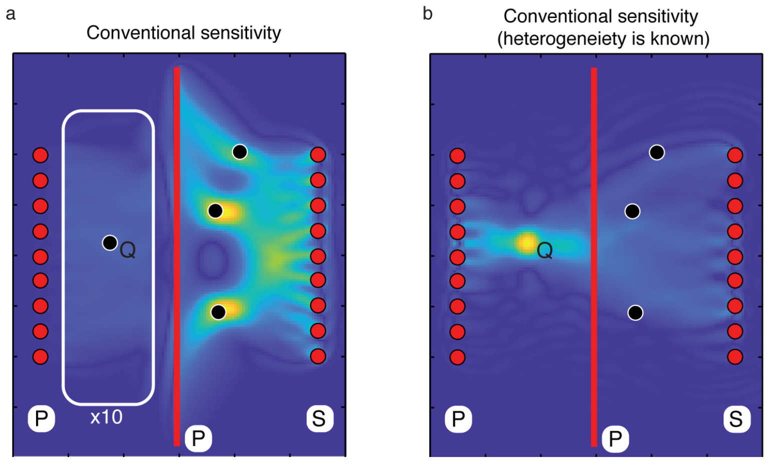

Supplementary Figure 1. The conventional sensitivity estimated using the same amount of data as in calculating the localized sensitivity (Fig. 2c). (a) Sensitivity assuming a homogeneous medium, and the reference receiver array used as additional observation points P. For better visibility, the sensitivity around Q is multiplied by 10. (b) Same as (a) but when the complete heterogeneity except for Q is known.

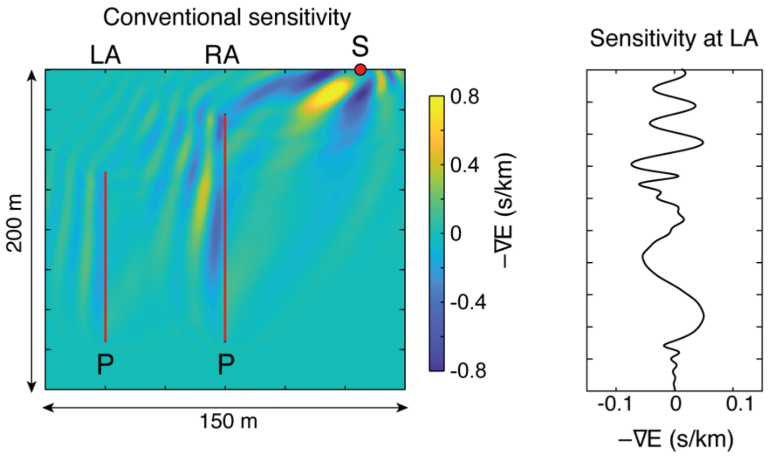

Supplementary Figure 2. The conventional sensitivity in the field experiments, using the same amount of data as in calculating the localized sensitivity (Fig. 4a). The sensitivity using the reference receiver array as additional observation points P is shown.

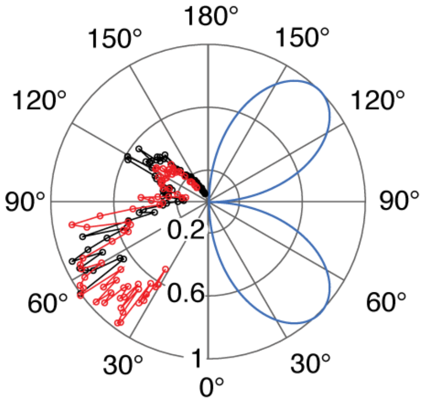

Supplementary Figure 3. Estimated radiation pattern (vertical component) in the independent waveform measurements using physical downhole sources. The results using the CRG at 52 m depth (black line) and at 98 m depth (red line) are shown. The blue line indicates the theoretical radiation pattern. The angle of 0° points the vertically downward direction.

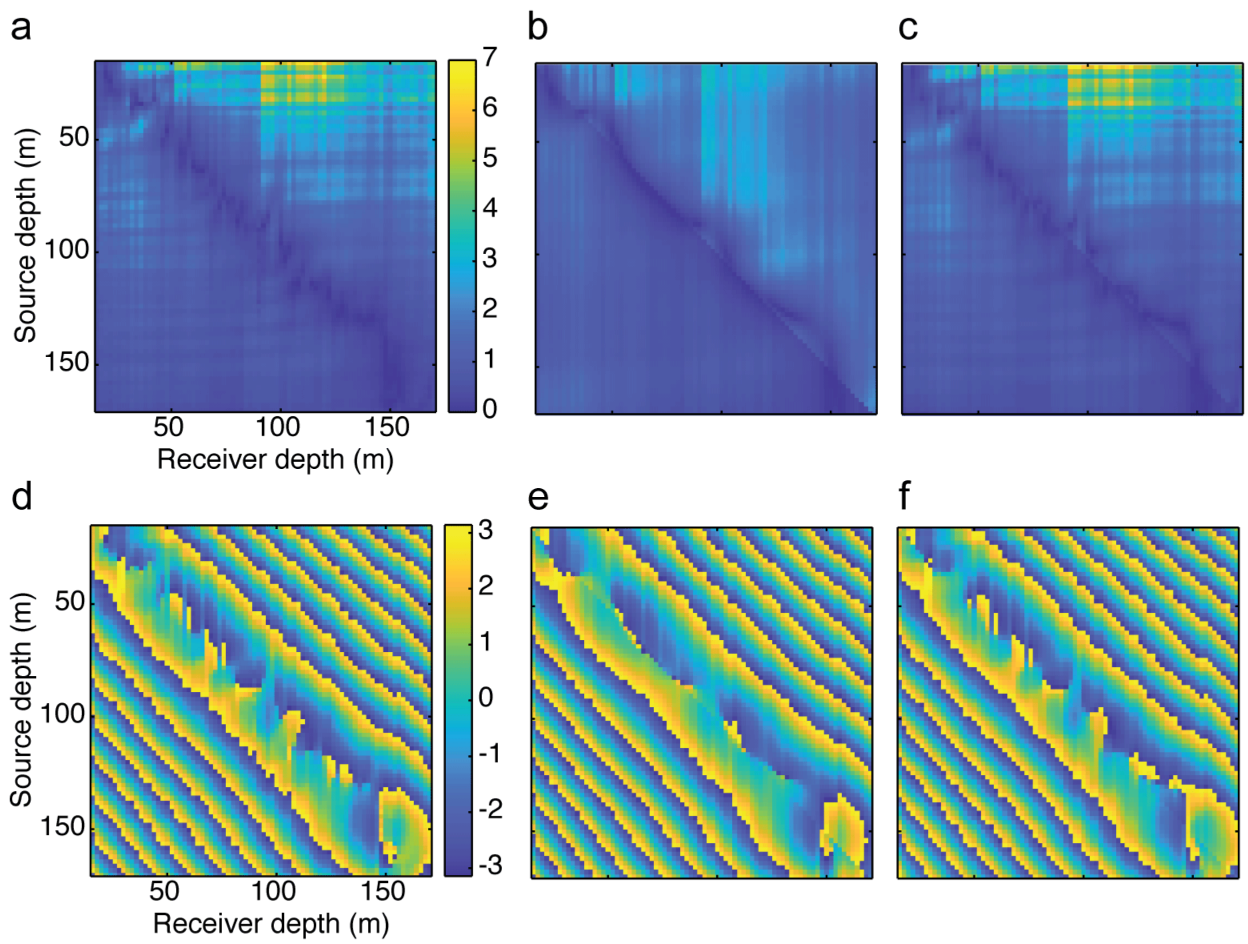

Supplementary Figure 4. Observed and predicted data at 120 Hz in the independent waveform measurements using physical downhole sources. (a) Amplitudes of the observed data, (b) predicted amplitudes using the initial velocity model, and (c) predicted amplitudes using the final velocity model. (d)-(f) are same as (a)-(c) but for the phase.

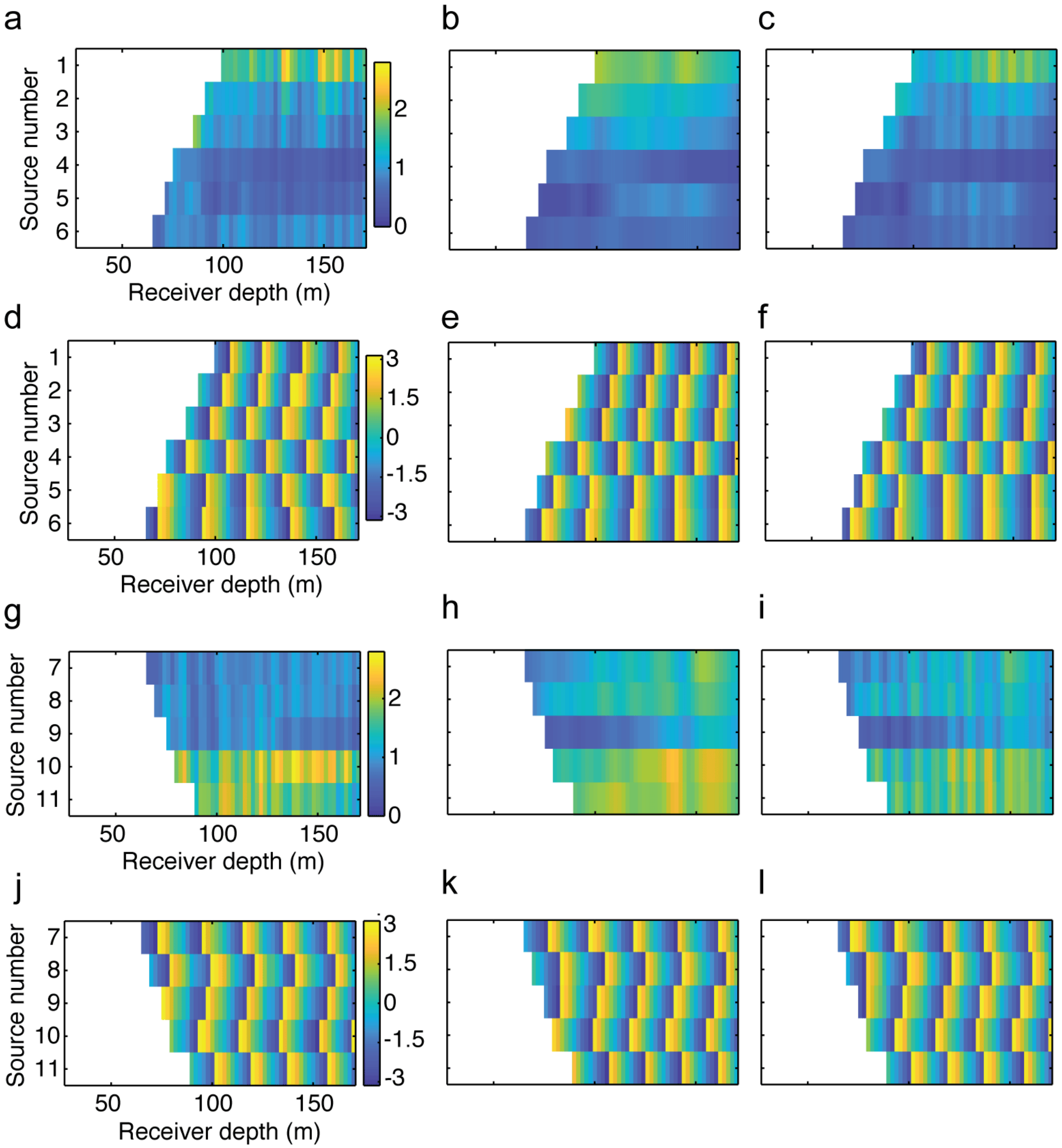

Supplementary Figure 5. Observed and predicted field data at 125 Hz in the proposed approach. (a) Amplitudes of observed data due to surface sources positioned to the left of LA. (b) Predicted amplitudes using the initial velocity model, and (c) predicted amplitudes using the final velocity model. (d)-(f) are same as (a)-(c) but for the phase. (g)-(l) are same as (a)-(f) but for data due to surface sources positioned to the right of RA.

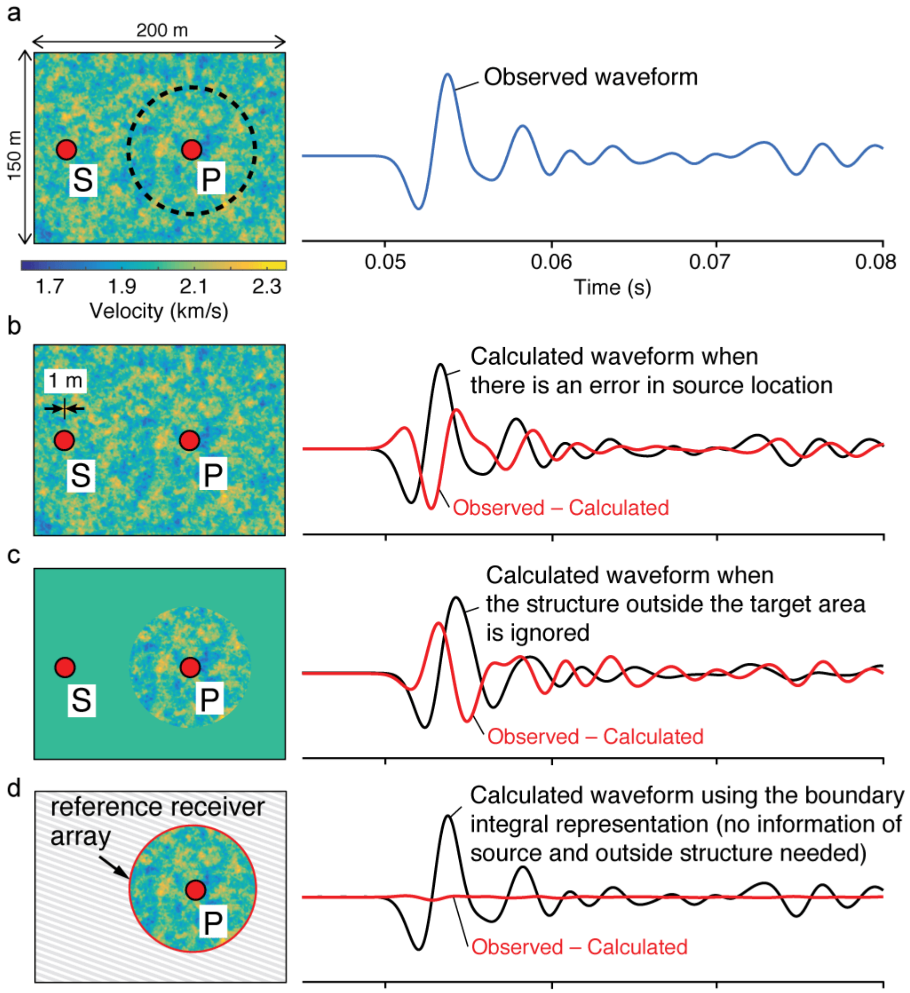

Supplementary Figure 6. 2D finite-difference (FD) numerical experiment: (a) At observation point P the waveform to a point source at S is recorded. The dashed circle marks the target area of interest. (b) Calculation using FD: The source and receiver locations and the intermediate velocity structure are input to compute the waveform ("calculation"). Here the waveform is calculated assuming the true velocity structure, but the source shifted by 1 m. The red line shows the calculation error (difference from the observation). (c) Calculation using FD: instead of true velocity, a constant velocity is assigned outside the target area, while the source is in its true location. (d) Calculation using the boundary integral representation: the input data consist of waveform observed at the reference receiver array surrounding the target area and the impulse response between P and the reference receiver array. In this case, no information of the structure outside the target area or the source location is necessary.

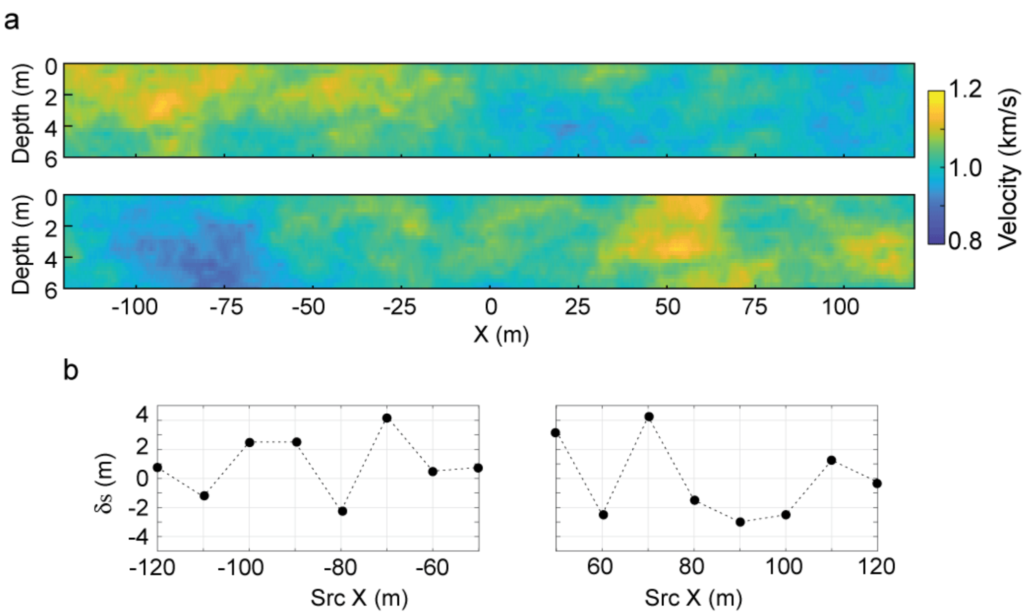

Supplementary Figure 7. Velocity structures in the numerical monitoring experiments: (a) The velocity models for the vadose zone (hatched area in Fig. 7a) in the baseline survey (top panel) and in the monitor survey (bottom panel). (b) The source location errors δs (difference in source locations between the baseline and the monitor surveys) fluctuate randomly. These errors at the baseline source locations to the left of LA (left panel) and to the right of RA (right panel) are shown.

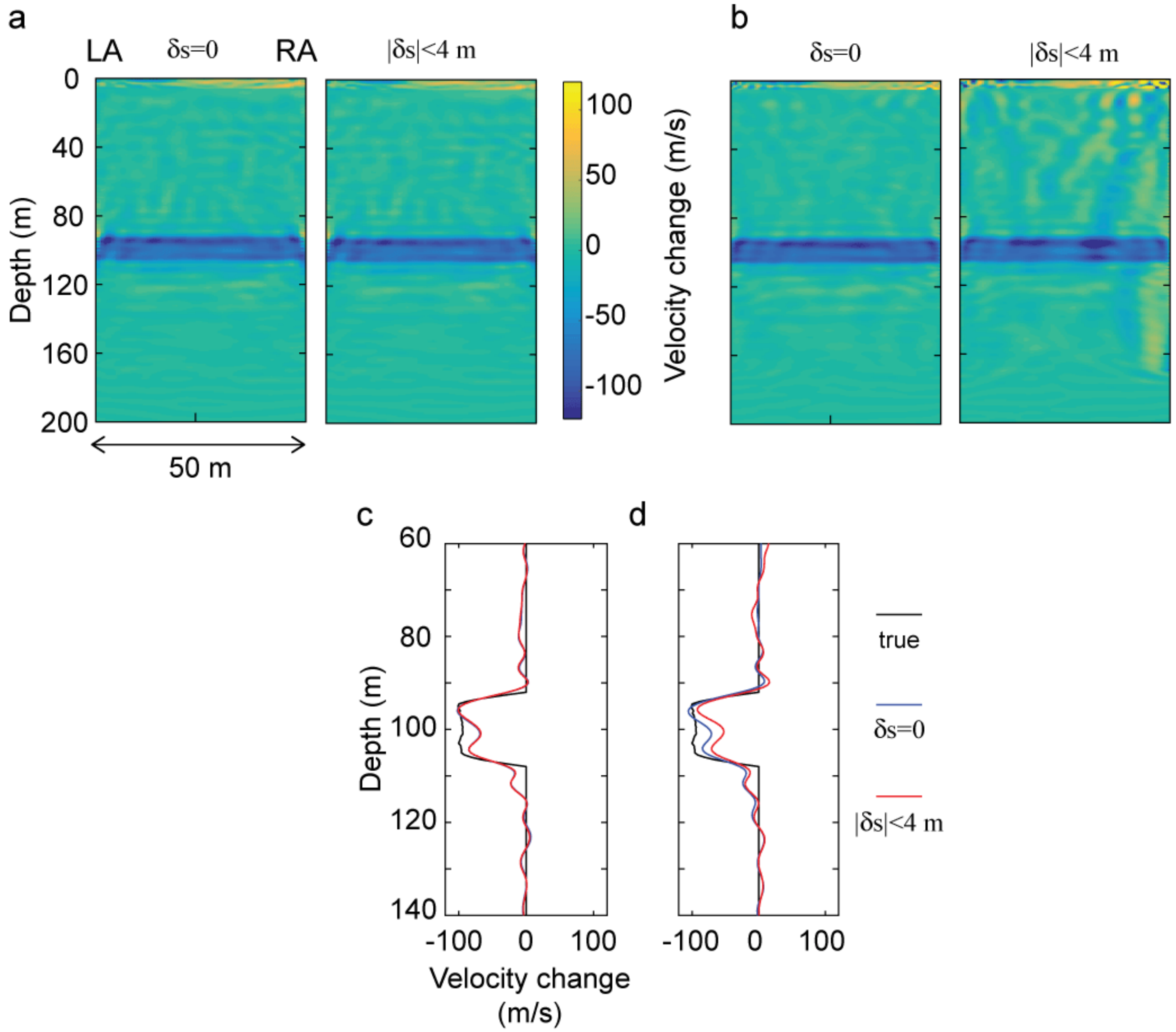

Supplementary Figure 8. Time-lapse changes at the target zone when the initial velocity model contains accurate prior information of the vadose zone (Fig. 7c): (a) True temporal changes. (b) Estimated temporal changes using the localized sensitivity for waveform tomography (this research). Results using data without source location errors (δs=0) and with source location errors (|δs| < 4 m) are shown. (c) Same as (b) but using the conventional sensitivity for the scattered waves. (d) Estimated velocity changes in the target area at x=0 m using the localized sensitivity, and (e) the same using the conventional sensitivity.

Supplementary References

1. Dankbaar, J. W. M. Vertical seismic profiling—separation of P-and S-waves. *Geophys. Prospect.* **35**, 803–814 (1987).
2. Ogura, K., Ohhashi, T., Osada, M. & Yoshimura, M. Design of a multidisk type downhole seismic source. in *SEG Technical Program Expanded Abstracts* 702–705 (1992).
3. Yokota, T. *et al.* Development of a Multi-disk type Borehole Seismic Source -Aiming at practical applications for oil field survey-. *Geophys. Explor.* **53**, 309–323 (2000).
4. Korenaga, J. *et al.* Crustal structure of the southeast Greenland margin from joint refraction and reflection seismic tomography. *J. Geophys. Res. Solid Earth* **105**, 21591–21614 (2000).
5. Humphreys, E. & Clayton, R. W. Adaptation of back projection tomography to seismic travel time problems. *J. Geophys. Res. Solid Earth* **93**, 1073–1085 (1988).
6. Lee, M. W. & Balch, A. H. Theoretical seismic wave radiation from a fluid-filled borehole. *Geophysics* **47**, 1308–1314 (1982).
7. Pratt, R. G. Seismic waveform inversion in the frequency domain, Part 1: Theory and verification in a physical scale model. *Geophysics* **64**, 888–901 (1999).
8. Pratt, R. & Shipp, R. Seismic waveform inversion in the frequency domain, Part 2: Fault delineation in sediments using crosshole data. *Geophysics* **64**, 902–914 (1999).
9. Aki, K. & Richards, P. G. *Quantitative Seismology second edition*. (University Science Books, 2002).
10. Shin, C. & Cha, Y. H. Waveform inversion in the Laplace-Fourier domain. *Geophys. J. Int.* **177**, 1067–1079 (2009).
11. Kamei, R. & Pratt, R. G. Inversion strategies for visco-acoustic waveform inversion. *Geophys. J. Int.* **194**, 859–884 (2013).
12. Irving, J. & Holliger, K. Geostatistical inversion of seismic and ground-penetrating radar reflection images: What can we actually resolve? *Geophys. Res. Lett.* **37**, (2010).
